# Lichen Planus Associated With an Orthopedic Implant: A Report of a Rare Case

**DOI:** 10.7759/cureus.101729

**Published:** 2026-01-17

**Authors:** Bradley Boman, Derrek M Giansiracusa, Lindsey Johnson, Jeffrey Dickman

**Affiliations:** 1 Internal Medicine, Southern Hills Hospital & Medical Center, Las Vegas, USA; 2 Dermatology, Midwestern University GME Consortium Phoenix Dermatology, Glendale, USA; 3 Dermatology, Omni Dermatology, Glendale, USA

**Keywords:** classic lichen planus, implant-related lichen planus, metal hypersensitivity, oral lichen planus, orthopedic implants

## Abstract

We report the case of a 56-year-old man who developed cutaneous lichen planus (LP) localized to the site of orthopedic hardware implantation one month following open reduction and internal fixation of an ankle fracture. The eruption was confined to the surgical scar and adjacent skin and demonstrated classic clinical features of LP, with no evidence of mucosal involvement. The patient denied exposure to known lichenoid triggers, including culprit medications, and hepatitis serologies were negative. Despite treatment with high-potency topical corticosteroids, systemic prednisone, and oral isotretinoin, the lesions exhibited only partial and transient improvement, with rapid recurrence upon discontinuation of therapy. Given the strict localization of disease to the implant site, absence of alternative triggers, and refractory clinical course, an implant-related immune-mediated reaction was suspected. Although lichenoid reactions to dental metals are well documented, reports of LP associated with orthopedic implants remain exceedingly rare. This case highlights the importance of considering implanted orthopedic hardware as a potential persistent antigenic stimulus in patients presenting with localized, treatment-resistant LP following surgical implantation.

## Introduction

Lichen planus (LP) is a chronic inflammatory dermatosis characterized by pruritic, polygonal, planar papules and plaques that display considerable morphologic variation [1]. Its pathogenesis involves immune dysregulation and associations with infections, genetic factors, and environmental exposures [1]. Lichenoid contact lesions caused by hypersensitivity to dental metals are well documented and often resolve with removal of the offending material [2]. Conversely, lichenoid reactions to orthopedic implants are exceedingly rare, with only two reported cases in the literature [3,4]. This highlights the need for further investigation into their immune-mediated mechanisms and the diagnostic and management challenges they present. Here, we present a case of LP developing shortly after open reduction and internal fixation (ORIF) of an ankle fracture. The clinical course was notably refractory to standard treatments, including topical and systemic steroids, likely due to ongoing antigen exposure from the implant. This case contributes to the limited literature on implant-associated LP and underscores the need for heightened clinical awareness and further research into its pathophysiology and management.

## Case presentation

A 56-year-old male presented to our dermatology clinic with a year-long history of a severely pruritic rash on the right lower extremity that persisted despite the use of over-the-counter topical treatments. He reported a history of an ankle fracture treated with ORIF approximately one year prior. He denied any significant past medical history. Of note, the patient’s rash began within a month of the procedure and has become progressively thicker and more pruritic.

**Figure 1:**
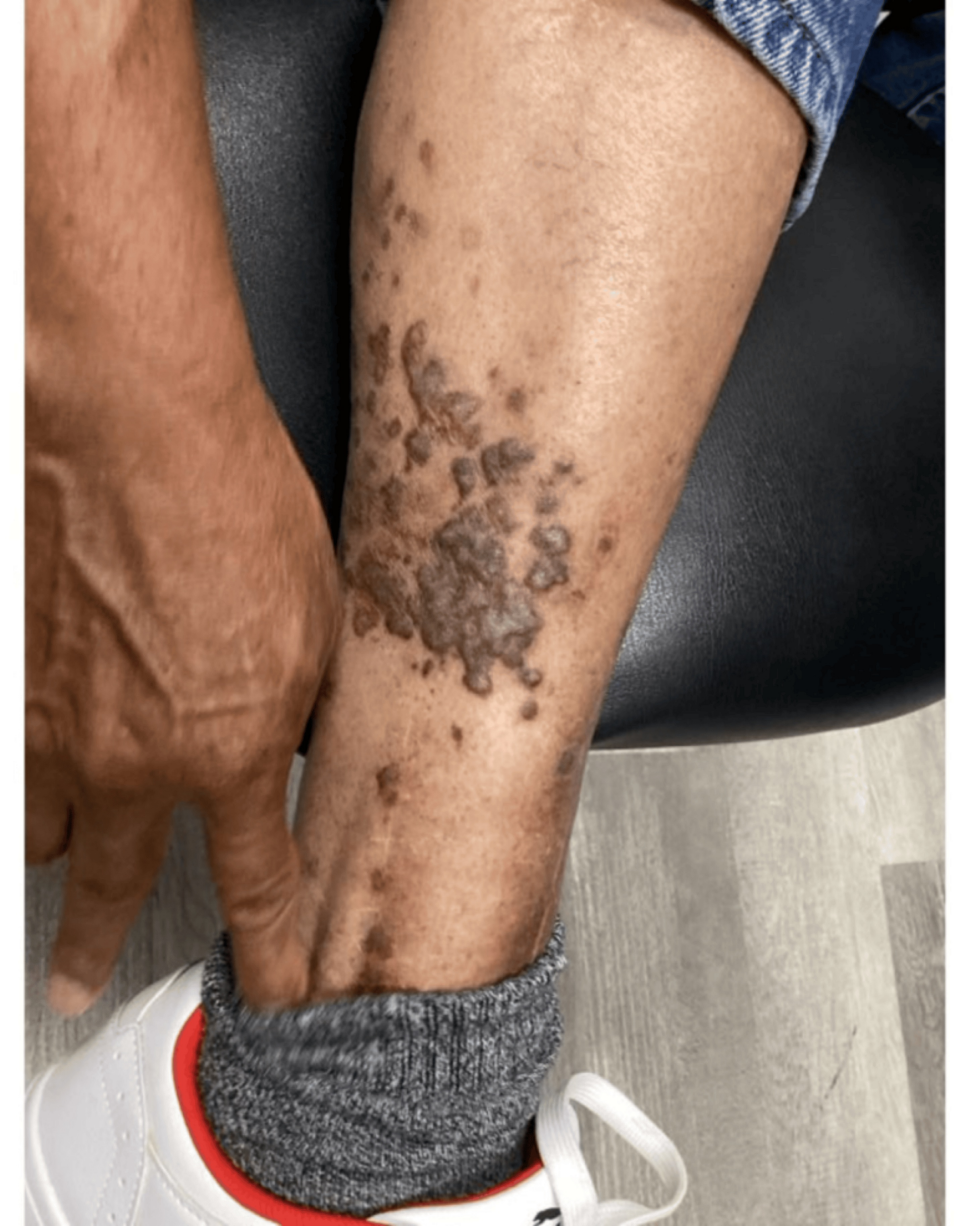
Initial presentation Lesions demonstrating violaceous and hyperpigmented, flat-topped papules coalescing into plaques surrounding the surgical site.

On physical examination, multiple violaceous and hyperpigmented polygonal, flat-topped papules with fine reticulated scale were noted. These lesions coalesced into a large plaque localized to the right lateral calf and surgical scar (Figure 1). There was no evidence of mucosal lesions in the mouth or genital areas. He denied use of common lichenoid triggers, including ACE inhibitors, beta-blockers, NSAIDs, antimalarials, thiazides, or TNF-alpha inhibitors. Hepatitis serologies were negative.

Given the classic clinical morphology of violaceous, polygonal, flat-topped papules with overlying Wickham striae, a clinical diagnosis of cutaneous LP was made. Biopsy was deferred as the presentation was characteristic and initial management would not have been altered. Initial treatment consisted of clobetasol 0.05% ointment applied twice daily and a four-week course of oral prednisone at 30 mg/day. At follow-up, the patient demonstrated notable improvement, with flattening of the plaques and reduced pruritus, though residual lesions remained.

**Figure 2:**
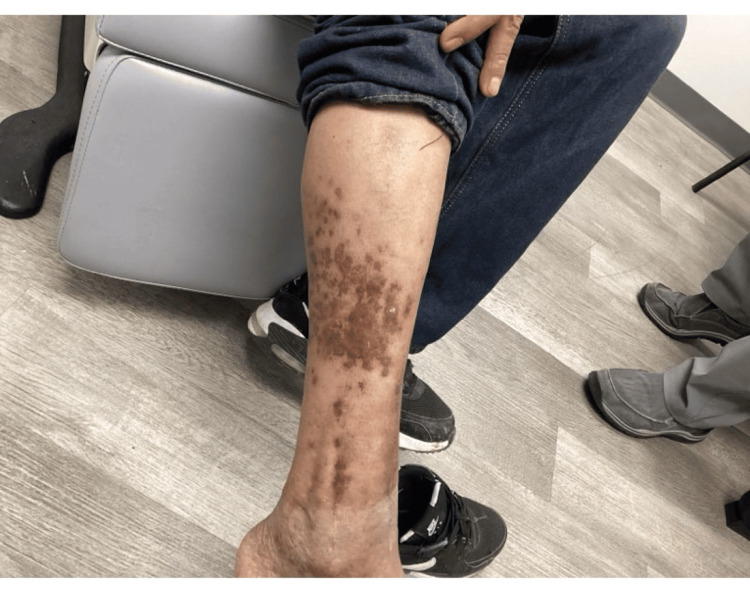
One-month posttreatment with oral isotretinoin therapy Lesions demonstrate significant flattening with residual post-inflammatory hyperpigmentation.

Due to the partial response, oral isotretinoin was initiated at 40 mg daily. After one month, the lesions showed further flattening with residual post-inflammatory hyperpigmentation (Figure 2). However, he discontinued treatment due to cost concerns and the burden of laboratory monitoring. One month after discontinuation, he returned to the clinic with a significant worsening of the rash. Oral metronidazole was prescribed; however, the patient was lost to follow-up. 

## Discussion

LP is an inflammatory skin condition that predominantly affects adults, with a prevalence of 0.98% in the general population [5]. It commonly presents as flat-topped, violaceous papules and plaques with fine white lines, known as Wickham striae. These lesions are often described by the “six P’s”: purple, pruritic, polygonal, planar, papules, and plaques [1]. While the eruption typically occurs on the extremities, less common patterns such as generalized, Blaschkoid, intertriginous, or dermatomal distributions have also been reported [1].

Clinical features can be characteristic, but biopsy with histologic evaluation is often pursued to confirm the diagnosis and exclude differential diagnoses [5]. Typical histologic findings include a band-like lymphocytic infiltrate obscuring the dermoepidermal junction with saw-tooth rete ridges, hyperorthokeratosis, irregular acanthosis, and wedge-shaped hypergranulosis [6]. Additional findings include Max-Joseph spaces, apoptotic keratinocytes forming Civatte bodies, and dermal melanophages [6].

At an immunologic level, LP is driven by CD8+ cytotoxic T cells, which target basal keratinocytes after antigen exposure, leading to apoptosis through Fas-FasL, TNF, and granzyme B pathways, amplified by CD4+ T cell cytokine signaling [7]. This immune response is thought to arise from a combination of genetic susceptibility, environmental triggers, and inflammatory mediators, including mast cells and pro-inflammatory cytokines [5]. Reported environmental triggers include viral infections, most notably hepatitis C, vaccines, alterations in the oral and skin microbiome, and certain medications such as angiotensin-converting enzyme inhibitors, thiazide diuretics, and antimalarials [5].

A well-documented trigger for oral LP (OLP) is exposure to foreign implanted materials, particularly dental implants. Amalgam restorations, in particular, have been shown to induce lichenoid lesions that closely resemble OLP [8]. These lesions often resolve following removal of the fillings, especially in patients with a strong clinical correlation and positive patch test results for mercury or amalgam [2,8].

Although the role of dental metals in precipitating oral lichenoid lesions is well documented, reports of lichenoid reactions associated with orthopedic hardware are exceedingly rare. Nickel, with a hypersensitivity rate of 18%, is a common component of such implants [3]. Other metals used in implants, including cobalt, vanadium, chromium, and titanium, have also been linked to hypersensitivity, and rates may rise further as their use continues to increase [3]. Despite the widespread implantation of these materials, only two cases of implant-associated LP have been described in the literature to date [3,4].

In the first case, a 64-year-old woman developed linear LP (LLP) seven months after receiving a chromium and cobalt implant for a femoral fracture [3,4]. Hypersensitivity to the implant metals was suggested, though patch testing was not performed. The second case involved a young male who developed LLP 15 days after receiving stainless steel implants for a leg fracture [3]. The lesions were localized to the surgical site, and patch testing was negative, ruling out hypersensitivity [3]. Although the eruptions occurred months after surgery, prosthesis-induced hypersensitivity remains a plausible mechanism, particularly given the absence of other risk factors or known triggers.

An alternative explanation to consider is the Koebner phenomenon, also known as the isomorphic response, which describes the development of lesions at sites of cutaneous injury that are clinically and histologically identical to a patient’s underlying dermatosis [9]. LP is a well-recognized Koebnerizing condition, along with psoriasis and vitiligo, and lesions may be induced by various forms of trauma, including surgical incisions, mechanical stress, friction, tattoos, and ultraviolet radiation [9]. Koebnerization, however, most commonly occurs in patients with pre-existing disease and typically manifests as new lesions at sites of trauma during an active disease course [9]. In contrast, our patient had no prior history of LP, and the eruption localized to the surgical site represented the initial manifestation of the disease. Furthermore, despite disease persistence over time, no lesions developed at distant or uninvolved sites, arguing against a generalized Koebner response. Taken together, these features make Koebnerization a less likely explanation for this presentation and suggest that trauma alone is insufficient to account for the localization and persistence of disease in this patient.

The increasing prevalence of orthopedic prosthesis implantation, driven by an aging population, has made the potential for implant-related complications, including skin conditions like LP, more relevant [4]. Orthopedic implants, particularly those made from materials such as chromium, cobalt, and molybdenum, have been shown to elevate metal concentrations in the bloodstream, as demonstrated by elevated serum and urine levels of chromium and cobalt in patients with implants [10]. This heightened concentration of metals could play a significant role in the development of LP, as these metals are known to trigger immune system activation, potentially through cytokine release, which may contribute to lesion formation [4].

Although patch testing to confirm metal hypersensitivity may not always be possible, the localization of LP lesions to the site of the implant suggests a potential link between the implant materials and the development of LP. In our case, the composition of the orthopedic implant could not be confirmed, as the patient was unable to recall the implant details or provide information to contact the operating orthopedic surgeon; additionally, patch testing to evaluate for metal hypersensitivity was not performed due to financial constraints. In cases where lesions develop over several months or years after implantation, such as the cases of LLP discussed earlier, it is plausible that the metals in the implants may act as long-term triggers, much like dental materials (e.g., amalgam and gold) that have been associated with OLP.

Treatment of LP depends on the extent and severity of the disease. First-line options include potent topical corticosteroids and topical calcineurin inhibitors; intralesional corticosteroid injections may be used for refractory lesions [5,6]. Systemic steroids, retinoids, and cyclosporine are additional first-line agents [5]. Second-line therapies involve sulfasalazine and phototherapy, particularly narrow-band UVB, which has demonstrated durable remissions and may be considered prior to systemic agents [5,6]. Other agents, including hydroxychloroquine, azathioprine, methotrexate, mycophenolate, and IL-12/23 biologics, are reserved for severe or resistant disease [5]. Antipruritic agents such as antihistamines provide symptomatic relief [5]. Emerging options include oral JAK inhibitors and dupilumab in select cases [6,7]. Most cutaneous cases resolve within one to two years, though relapses and post-inflammatory hyperpigmentation are common [1,5].

Our patient’s course was notable for its refractory nature compared to typical cutaneous LP, which often responds to high-potency topical corticosteroids and short courses of systemic therapy. In this case, only partial and temporary improvement was observed following treatment with oral corticosteroids and isotretinoin, and relapse occurred quickly upon discontinuation of treatment. We suspect that the orthopedic hardware acted as a persistent antigenic stimulus, perpetuating local immune activation and making the eruption more resistant to conventional therapies.

As the long-term biological effects of elevated metal concentrations remain uncertain, further research is needed to explore the exact mechanisms by which these materials might contribute to the development of LP and other dermatologic conditions. In patients with persistent or unusually treatment-resistant LP localized to a surgical site, clinicians should consider implant-related hypersensitivity in the differential, as identifying and addressing the underlying trigger may be key to achieving lasting disease control.

## Conclusions

LP associated with orthopedic implant is an uncommon dermatosis presenting with typical LP lesions localized to an orthopedic surgical site. These lesions may be refractory to topical medications and require progression to systemic therapies to obtain lasting disease control. We suspect the recalcitrant nature of these lesions may be secondary to the metal implant acting as a chronic antigenic stimulus perpetuating an immune response. More research is needed to elucidate the underlying mechanisms involved in implant-related LP, and we encourage further reporting of these rare and unique cases.
